# Aquaporins as Prognostic Biomarker in Prostate Cancer

**DOI:** 10.3390/cancers15020331

**Published:** 2023-01-04

**Authors:** Prem Prakash Kushwaha, Shiv Verma, Sanjay Gupta

**Affiliations:** 1 Department of Urology, School of Medicine, Case Western Reserve University, Cleveland, OH 44106, USA; 2 The Urology Institute, University Hospitals Cleveland Medical Center, Cleveland, OH 44106, USA; 3 Department of Pathology, Case Western Reserve University, Cleveland, OH 44106, USA; 4 Department of Pharmacology, Case Western Reserve University, Cleveland, OH 44106, USA; 5 Department of Nutrition, Case Western Reserve University, Cleveland, OH 44106, USA; 6 Division of General Medical Sciences, Case Comprehensive Cancer Center, Cleveland, OH 44106, USA

**Keywords:** aquaporin, prognostic biomarkers, prostate cancer, membrane water channels, signaling pathway

## Abstract

**Simple Summary:**

Aquaporins (AQPs) are transmembrane channel proteins that primarily transport water across the cellular membranes. AQPs have been found to be overexpressed in various human cancers, including prostate cancer. Clinical data suggest ideal prospects for AQPs as biomarkers. This review article mainly focuses on the opportunities for the development of AQPs as prognostic markers in prostate cancer.

**Abstract:**

Prostate cancer is a complex heterogeneous disease that affects millions of males worldwide. Despite rapid advances in molecular biology and innovation in technology, few biomarkers have been forthcoming in prostate cancer. The currently available biomarkers for the prognosis of prostate cancer are inadequate and face challenges, thus having limited clinical utility. To date, there are a number of prognostic and predictive biomarkers identified for prostate cancer but lack specificity and sensitivity to guide clinical decision making. There is still tremendous scope for specific biomarkers to understand the natural history and complex biology of this heterogeneous disease, and to identify early treatment responses. Accumulative studies indicate that aquaporins (AQPs) a family of membrane water channels may serve as a prognostic biomarker for prostate cancer in monitoring disease advancement. In the present review, we discuss the existing prostate cancer biomarkers, their limitations, and aquaporins as a prospective biomarker of prognostic significance in prostate cancer.

## 1. Introduction

Prostate cancer (PC) is a biologically heterogeneous disease and the most common cancer among males. According to the American Cancer Society, in the year 2022, approximately 268,490 new cases and 34,500 deaths occurred in the United States alone [1]. Inflammation, genetic modifications, and increased cellular proliferation are major critical factors for the initiation of prostate cancer [2]. Prostate cancer in humans exhibits a unique spectrum of features: multifocality, heterogeneity, variable clinical progression, propensity to metastasize to bone, and the emergence of androgen-independent disease forms [2]. Long-term clinical outcomes for men can vary greatly, even when they are diagnosed with organ-confined disease [3]. There is substantial variability among patients and within tumors in terms of histologic and molecular characteristics [4]. Progress in the treatment of prostate cancer has also been hindered by the fact that histologically identical cancers in different patients may exhibit widely variant biologic behavior [4]. These challenges pose major implications in the clinical management of prostate cancer.

The current prognosis of prostate cancer is highly variable and depends on the degree of cancer and its stage at the time of diagnosis. Acceptance of screening based upon the measurement of prostate-specific antigen (PSA) has led to earlier detection of prostate cancer however lack of specificity to detect a significant number of PSA-negative tumors limits its detection efficacy [5]. In fact, PSA has been shown to test positive for common confounders such as benign prostatic hyperplasia [6]. As a result, over 90% of the low-risk Gleason score (GS) (6 or less) prostate cancer patients receive aggressive treatment to avoid potential cancer-related deaths [7]. This has led to concerns regarding overdiagnosis and the overtreatment of prostate cancer [8]. Therefore, there is an urgent need to identify novel prognostic biomarkers to determine those patients that are at higher risk for progression and might benefit from more aggressive treatment, and patients that might be spared from unnecessary and potentially harmful interventions.

Recent advancements in genomic and proteomic techniques combined with progress in bioinformatics demonstrate great promise in the identification of several new biomarkers of diagnostic and prognostic value in prostate cancer [9]. In the present review, we discuss the existing prognostic biomarkers of prostate cancer, their limitations, and aquaporins as a prospective biomarker of prognostic significance in prostate cancer.

## 2. Prognostic Biomarkers and Their Limitations in Prostate Cancer

Prognostic biomarkers aim to evaluate objectively patient’s overall outcome and are essential to provide important clinical decisions for prostate cancer patients. Besides predicting clinical progression, it is usually considered that such prognostic biomarkers also provide valuable information about disease mechanisms and the underlying molecular processes. Although, prognostic biomarkers, even after a long history of research, are still not recommended for use in clinical settings although quite a few of them are considered potential candidates. However, with no data currently available about their clinical relevance in the long term, also prospective validation is lacking. A list of commercially available prognostic biomarkers and their limitations are listed in Table 1.

There are a number of prognostic tests available for commercial gene expression signatures. These subsets of biomarkers assist clinicians in discriminating against aggressive vs. indolent prostate tumors. The ConfirmMDx, Prostate Core Mitomic Test (PCMT), phosphatase and tensin homolog (*PTEN*) gene, TMPRSS2-ERG gene fusion, and ProMark are few commercially available tests [24]. Epigenetic-based ConfirmMDx detects epigenetic field effect associated with cancerization in the DNA [25]. It is useful for identifying patients with true negative biopsy results from those with occult cancers. Prostate Core Mitomic Test detects malignant cells in normal-appearing prostate tissue across a wide area by identifying a large-scale depletion in mitochondrial DNA associated with undiagnosed prostate cancer [26]. Prostate cancers with the TMPRSS2-ERG gene fusion account for 40 to 80% of total cases [27]. Clinically significant prostate cancer is associated with high urine TMPRSS2-ERG levels based on Epstein criteria that stratify disease aggressiveness using PSA density and biopsy characteristics including the percentage of normal and tumor prostate tissue detected, Gleason score, and the number of tumor cores [27]. A defective tumor suppressor *PTEN* gene, involved in the regulation of the cell cycle, steadily correlates with a poor prognosis in prostate cancer [28]. Accumulative evidence showed a positive association between deletion of the *PTEN* gene and progression risk, higher Gleason grades, and recurrence after therapy [28]. Aside from this, it has been linked to advanced localized or metastatic disease as well as death [28]. ProMark is another biopsy-based prostate cancer test that quantifies biomarker expression and classifies patients’ tumors based on analysis of immunofluorescent imaging [29]. On the basis of formalin-fixed, paraffin-embedded tissue data from a clinical validation study, ProMark is capable of differentiating indolent from aggressive disease [29].

Several genetic tests of biopsy specimens are now commercially available for risk stratification of patients with prostate cancer such as Prolaris, Oncotype DX and Decipher [30]. The Prolaris test evaluates the expression of 31 cell-cycle–related genes and 15 housekeeping genes [31]. The results are represented as a cell-cycle progression (CCP) score. Combining standard clinicopathologic parameters with CCP score provides predictive genomic data regarding prostate cancer-specific progression and disease-specific mortality. This assay has been validated in multiple cohorts. Based on validation studies, Prolaris can identify patients with low risk who can be treated conservatively and those with a high-risk disease that may take advantage from earlier definitive treatment [32]. The Oncotype Dx test evaluates the expression of 12 cancer-related genes and five housekeeping genes [33]. The results are represented as a Genomic Prostate Score (GPS). In men harboring very low, low, and low-intermediate risk prostate cancer, in a prospective study, Oncotype DX has been demonstrated to predict adverse pathology based on biopsy [33]. Beyond clinical and pathological measures, the GPS offers independent predictive information. Using biopsy tumor volumes from very small biopsy specimens, GPS assesses underlying biology to determine disease aggressiveness with greater accuracy, considering tumor heterogeneity and undersampling [34]. The Decipher test evaluates 22 cancer-related genes. The results are represented as a Genomic Classifier score intended to help predict the risk of metastasis after radical prostatectomy [35]. A high-risk surgical cohort has shown that this assay is independently prognostic of prostate cancer death. These genomic-based tests demonstrate rigorous quality criteria including reproducibility, linearity, analytical accuracy, precision, and are reliable prognostic tools for the prediction of biochemical recurrence or prostate cancer-specific survival albeit their systematic use in prostate cancer is currently not recommended due to insufficient evidence.

## 3. Aquaporin Family and Their Function in Normal and Cancer Pathophysiology

**Figure 1:**
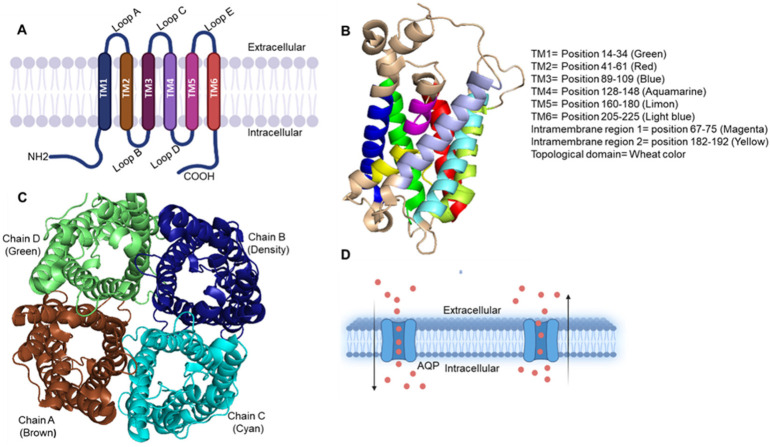
(**A**) Representation of three-dimensional structure of aquaporin and its topology. The illustration shows AQP protein structure, a membrane-bound protein composed of six transmembrane helices and has both the carboxyl and amino terminus regions positioned in the cytoplasm. All six transmembrane helices show connectivity with five loops named as A–E. (**B**) Monomer structure (side view) of AQP visualized by PyMOL software. It shows six transmembrane, two intramembrane and all topological domains present in the AQP with defined position and color. (**C**) Tetramer structure (top view and active form) of AQP, which is made up of four AQP monomer chains A–D (represented with different color) which in altogether forms pore in the middle for solute transfer passively. PyMOL software was used to visualize the figure. (**D**) Passive transport of water molecules through aquaporins across the membrane.

Aquaporin families are small-size (24–30 kDa) pore-forming integral membrane proteins. The Aquaporin gene encodes six bilayer spanning domains integral membrane protein that forms water channels and permits osmotic gradient-mediated (passive) transport (Figure 1).

To date, thirteen isoforms (AQP0–AQP12) of the AQP family have been recognized in humans and categorized into three subfamilies [36]. The first subfamily of aquaporin is water-selective pore-forming proteins, which are known as classical aquaporins including AQP0, AQP1, AQP2, AQP4, AQP5, AQP6 and AQP8 [37]. Numerous studies have been conducted on this subfamily of AQPs, which have been useful for better understanding the role they might play in physiological and pathophysiological conditions [38]. Recent studies revealed that AQP6 and AQP8 are unorthodox aquaporins since AQP6 is highly water permeable and AQP8 has unique phylogenetics setting it apart from other aquaporins [39]. In mice lacking AQP1, urine concentration is greatly impaired because it cannot concentrate in the descending vasa recta, the descending limb of the loop of Henle, and the epithelium of the renal proximal tubule [40]. AQP4 is the primary water channel expressed in astrocytes throughout the central nervous system [40,41,42]. This protein is known to participate in water transport between the brain and spinal cord, in neuroexcitation and in astrocyte migration following injury [43]. Studies reported the abundance of AQP2 and AQP4 expression mainly in kidney-collecting duct epithelial cells [44]. The second subfamily of aquaporins is denoted by aquaglyceroporins that are permeable to small uncharged molecules, water, and other molecules such as urea, ammonia, and glycerol [45]. Aquaglyceroporins play an important role in metalloid homeostasis and facilitate arsenite and antimonite diffusion [46]. A comparison of amino acid sequence alignments can distinguish the aquaporins from aquaglyceroporins (AQP3, AQP7, AQP9, and AQP10) [47]. First cloned mammalian aquaglyceroporin was AQP3 which was able to facilitate water and glycerol transportation [48], however in xenopus oocytes, AQP7, AQP9, and AQP10 transport water, glycerol, and urea [49]. Oocytes also allow the flow of a wide range of solutes through AQP9 [50]. Glycerol and urea are transported by aquaglyceroporins, which are still poorly understood. Unlike the first two subfamilies, the third aquaporin subfamily is characterized by low conservation of amino acid sequences around the asparagine-proline-alanine (NPA) boxes [51]. In mammals, there are only two super aquaporins, named AQP11 and AQP12 [51]. With a homology of less than 20%, these two AQPs appear to belong to a supergene family of AQPs, as their NPA boxes are highly different from those of other classical AQPs [52]. At present, little is known about the structure and function of AQP11 and AQP12.

Upregulation of AQPs has been demonstrated in many tumor types, such as breast, prostate, lungs, brain, liver, cervical, ovarian, skin, renal, stomach, esophageal, thyroid, and colorectal cancer [53]. There is evidence to suggest that AQPs play a crucial role in cancer metastasis and progression. Silencing of AQP1 in mice has been shown to reduce tumor growth and angiogenesis [54]. Studies reported that increased angiogenesis induced by AQP1 through endothelial cell stimulation is via estrogen receptors [55]. Additionally, AQP3 may facilitate glycerol transport into the mammary gland, fueling growth demands by increasing intracellular ATP [56]. Furthermore, AQP4 knockdown inhibits cell invasion in human glioma cells, while AQP8 overexpression promotes cervical cancer cell invasion [57,58]. Researchers have also demonstrated that co-expression of AQP3 and AQP5 is associated with aggressive tumor progression as well as poor outcomes in esophageal squamous cell carcinoma [59]. Moreover, it has also been shown that silencing AQP3 improves the effectiveness of cryotherapy for prostate cancer [60]. AQP5 and AQP9 are associated with drug resistance in colorectal chemotherapy [61]. Several studies reported the prognostic potential of AQPs in breast cancer [62], renal cell carcinoma [63], ovarian cancer [64], and lung adenocarcinoma [65]. In this sequence, AQPs could be proposed as prognostic biomarker(s) for prostate cancer.

## 4. Aquaporin Expression in Prostate Cancer

Aquaporin 1 (AQP1) regulates the permeability of epithelial and endothelial barriers by assisting water movement across cell membranes. Different levels of AQP1 expression has been shown to correlate with tumor stage in cancer patients [66]. A study reported that AQP1 facilitates interstitial fluid pressure and high vascular permeability in the carcinoma of the colon, brain, pancreas, and breast [67]. In addition, AQP1 is involved in the effusion or edema fluid development that stimulates tumor angiogenesis [67]. A study was conducted for comparative abundance and distribution analysis of AQP1 in tumor and normal tissue of the prostate. The outcome of the study demonstrated that AQP1 was expressed in capillary endothelia of all normal tissues and slightly higher in microvascular structures. This suggests that overexpression of AQP1 may be a consequence of angiogenesis and perform a significant role in tumor edema formation or clearance [67]. Another study examined specimens from benign prostate hyperplasia and prostate cancer and demonstrated that AQP1 is majorly expressed in venules and capillaries of the prostate [68]. A clinical trial (NCT00851994) was conducted to evaluate the specificity and sensitivity of AQP1 concentration to diagnose the clear cell or papillary renal cell carcinoma (RCC) by comparing urine AQP1 concentrations in RCC, bladder cancer, non-cancer renal masses, and prostate cancer patients [69]. The results demonstrate that AQP1 could be a suitable biomarker having excellent specificity and sensitivity in the urine sample of RCC patients. Another study reported that cell density-induced pericellular hypoxia and cobalt (II) chloride (CoCl(2))-induced hypoxia phosphorylates p38 mitogen-activated protein kinase (MAPK) which enhance AQP1 expression [66]. Furthermore, protein kinase C (PKC) and intracellular calcium ion (Ca^2+^) activates p38 MAPK pathway enhancing AQP1 expression. Induction of AQP1 expression is also dependent on the lower oxygen (O2) levels [66]. One of the study highlighted that certain secretory proteins indirectly induce AQP1 expression [70]. Other studies reported that malnutrition increases the prevalence of non-communicable chronic diseases such as cancer [71,72]. In another study, researchers found increased expression of AQP1 and oxidative stress levels in malnourished rat model together contribute to prostate carcinogenesis in offspring [73]. A study assessed the AQP1 expression and their clinico-pathological significance in prostate adenocarcinoma. Tissue microarray analysis of paired malignant and benign prostatic tissues revealed higher expression of AQP1 in 17.2% specimens with both low and high Gleason scores. Positive association of AQP1 overexpression and higher Gleason score was associated with higher pathologic stages, and biochemical recurrence [74]. A transcriptomic analysis revealed that increased transcript levels of AQP1 was significantly associated with poor survival of prostate cancer patients [58].

Aquaporin 3 (AQP3) channel proteins transport water, nonionic small solutes such as urea and glycerol and other small solutes across the cell membrane [75]. Aberrant AQP3 expression has been reported in colon cancer [76], lung cancer [77,78], and esophageal and oral squamous cell carcinoma [79]. A cDNA microarray-based study demonstrates that prostate cancer cells exhibit overexpression of AQP3 protein [80]. Chen et al. (2015) showed that AQP3 silencing by small interfering RNA (siRNA) inhibited motility and invasiveness in prostate cancer cells through a reduction in extracellular signal-regulated kinase (ERK) ½ activation [80]. The study also showed that AQP3 upregulates the matrix metalloproteinase 3 expression and its secretion in prostate cancer through activation of the ERK signal pathway [80]. Subcellular localization of AQP3 in normal human prostate cells is restricted to the cell membrane whereas in prostate cancer, AQP3 is frequently located in the cytoplasm [81]. Furthermore, localization of AQP3 was limited to the basolateral cell membranes in the normal epithelia of the prostate. However, in cancer, AQP3 expression was not observed on the cell membranes. Nejsum and Nelson, (2007) confirmed that AQP3 co-localizes with E-cadherin during the early stages of cell–cell contact formation [82]. AQP3-E-cadherin co-localization depends upon the level of E-cadherin and increased E-cadherin levels simultaneously increase AQP3 expression in the plasma membranes of prostate epithelial cells [83]. Another study reported that the knockdown of RAS such as proto-oncogene A (RalA) facilitates increased AQP3 expression onto the plasma membrane [83]. The absence of RalA suppressed cell motility and invasion in prostate cancer cells. This mechanistic study revealed that redistribution of AQP3 in prostate cancer occurs through RalA/PKA/cAMP signaling pathways [83]. The role of hypoxia in AQP3 expression and its cellular localization reveals that both chronic and acute hypoxia plays a crucial role in the adaptation of AQP3 in the plasma membrane [84]. Khan et al. (2021) demonstrated that regulation of the AQP3 gene occurs by estrogen response elements (ERE) in prostate cancer [85].

Aquaporin 5 (AQP5) is an androgen-regulated member of a family of small hydrophobic integral transmembrane water channel proteins regulating cellular water homeostasis and growth signaling. AQP5 is overexpressed in colon cancer [86,87], lung cancer [88], cervical cancer [89], leukemia [90], esophageal cancer [68], ovarian cancer [91], and hepatic cancer [92]. Research revealed that AQP5 expression is associated with *PTEN* deletion and ERG positivity. AQP5 positivity was observed in ERG-positive (15.5%), ERG-negative (5.8%), with *PTEN* deletion (14.7%) and without *PTEN* deletion (9.4%) prostate cancers. Notably, both AQP5 positivity and AQP5 negativity were associated with disease aggressiveness [93]. The clinical significance of AQP5 and its correlation with key genomic alterations in prostate cancer was investigated by Pust et al. (2016) on a tissue microarray containing 12,427 prostate tumors [93]. The study revealed lower expression of AQP5 in normal prostate epithelium, whereas in prostate cancer, its expression showed a dichotomous pattern. Immunostaining showed 25.0% negative, 32.5% weak, 32.5% moderate, and 10.0% strong AQP5 staining in 10,239 interpretable tumors. Furthermore, another research group attempted to propose AQP5 as a prognostic biomarker by evaluating AQP5 expression in 60 prostate cancer specimens and prostate cancer cell lines. The result showed that 31.7% (19) patients exhibited high levels of AQP5 expression, 50.0% (30) showed intermediate, and 18.3% (11) showed absence of AQP5. Increased AQP5 expression frequently accompanies gene amplification associated with TNM stage and lymph node metastasis. However, the association between tumor size and age with AQP5 expression was not noteworthy. A positive correlation between circulating tumor cells and negative cumulative survival rate was observed with AQP5 expression in prostate cancer patients [94]. On the contrary, the transcriptomic analysis revealed that increased AQP5 mRNA levels were highly associated with poor survival [58]. A study performed by Park and Yoon (2017) showed that AQP5 expression negatively correlate with neoplastic and non-neoplastic tissue and established no correlation with clinicopathological parameters [74].

Aquaporin 9 (AQP9) overexpression has been linked with carcinoma of the kidneys, liver, lungs, colorectum, brain, ovarian, prostate, and liver [95]. AQP9 is expressed in the cytoplasm of prostate epithelial cells both in the benign and malignant stages [48]. Transcriptomic analysis revealed that elevated AQP9 mRNA levels were positively associated with poor survival [58]. AQP9 gene regulation occurs by estrogen response elements (ERE) in prostate cancer [85]. A research group evaluated and confirmed the androgen-dependent upregulation of AQP9 in prostate cancer [96]. A study explored the androgen-independent expression of AQP9 in prostate cancer PC3 cells, and in prostate cancer samples and adjacent cancer tissues [97]. AQP9 gene silencing in PC3 cells inhibits proliferation. Furthermore, the absence of AQP9 decreases anti-apoptotic protein Bcl-2 expression and increases apoptotic protein expression (cleaved caspase 3 and Bax) suggesting that AQP9 regulates apoptosis in prostate cancer. AQP9 expression also affects the motility and invasiveness of prostate cancer cells. In-depth analysis revealed that the absence of AQP9 led to reduced ERK1/2 phosphorylation which suggests that activation of the ERK pathway requires AQP9 channel proteins [97].

Several others AQPs such as AQP4, AQP7, AQP8, AQP10 and AQP11 are also overexpressed in prostate cancer cells and in both benign and malignant human prostate tissue at the transcript level. Immunofluorescence microscopy confirmed AQP4 and AQP7 protein expression in human prostate tissue [98]. A list of AQPs that have been expressed in prostate cancer is shown in Table 2.

## 5. Do Aquaporins Serve as Prognostic Biomarkers for Prostate Cancer?

**Figure 2:**
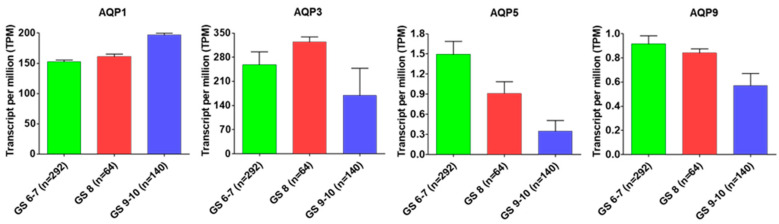
AQPs expression status with Gleason score. AQP1, AQP3, AQP5 and AQP9 expression status with Gleason score in prostate adenocarcinoma (PRAD) based on the cancer genome atlas (TCGA) dataset.

The present scenario of prostate cancer prognosis involves various biomarkers which can serve in the primary detection of prostate cancer however their role in discrimination against malignant progression is still untrustworthy. In this direction, research studies indicating that aquaporins have capability to compete as a successful biomarker for prognosis of prostate cancer. Aquaporins play important roles in the maintenance of water balance, including cellular migration, cellular expansion, and cellular adhesion facilitation together with a significant association between aquaporins expression and tumor grade [53]. Several research findings indicate that AQPs such as AQP1, AQP2, AQP3, and AQP5 have been confirmed as useful biomarkers for hepatocellular carcinoma, breast cancer, lung adenocarcinoma and colorectal cancer [53,61,85]. However, AQPs as prognostic biomarker-related research studies are limited in prostate cancer. For the first time, Park and Yoon (2017) reported that AQP1 can perform as a prognostic factor for biochemical recurrence in prostate adenocarcinoma [74]. In another study, researchers measured AQP5 expression in prostate cancer tissues and cell lines and established its expression is highly correlative with tumor (T), nodes (N), and metastases (M) (TNM) stage [94]. Even though AQPs including AQP1, AQP3, AQP5 and AQP9 have exhibited significant roles that contribute to prostate cancer prognosis, very little progress has been made in identifying AQPs as biomarker(s) for their use in prostate cancer. OncoPrint data analysis of prostate cancer patients further uncovers the possibilities of AQP1, AQP3, AQP5 and AQP9 being developed as prognostic biomarkers for prostate cancer (Figure 2).

Results revealed that AQP1 exhibited a positive correlation with the Gleason score; and with a progressive increase in the Gleason score AQP1 expression increases in prostate tumor. AQP3 expression also positively correlate with tumor progression, but in advance-stage prostate tumors with high Gleason score (GS, 9–10), a decrease was observed in its expression. Furthermore, a progressive decrease in the expression of AQP5 and AQP9 was observed during prostate cancer progression that negatively correlates with their expression.

**Figure 3:**
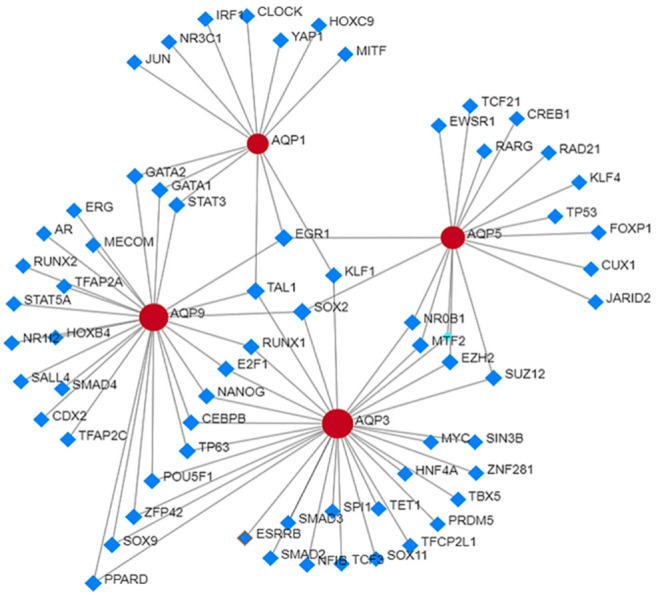
Protein–protein interaction analysis of AQPs with other proteins in prostate cancer.

Studies on protein–protein interaction between AQP1, AQP3, AQP5, and AQP9 have identified some key molecules that play a critical role in driving cellular processes including cell–cell adhesion, proliferation, migration, angiogenesis, and stemness. Protein–protein interaction search revealed that major aquaporins such as AQP1, AQP3, AQP5 and AQP9 interact with other proteins including androgen receptor (AR), signal transducer and activator of transcription (STATs) (STAT5A, STAT3), sma- and mad- related protein (SMADs) (SMAD2, SMAD3 SMAD4), stemness-related transcription factors such as SOX2, SOX9, SOX11, NANOG, MYC, epigenetic modifiers such as enhancer of zeste 2 polycomb repressive complex 2 (EZH2), suppressor of zeste 12 protein homolog (SUZ12), kruppel-like factors (KLF1, KLF4) and transcriptomic regulators including E2F1, cyclic AMP-responsive element-binding protein 1 (CREB1), zinc finger protein 281 (ZNF281), Yes-associated protein 1 (YAP1). Some of these proteins including AR, SMAD, NANOG, EZH2, and KLF have demonstrated prognostic significance in advance-stage prostate cancer. Rebello et al. (2021) reviewed the role of AR in prostate cancer progression [2]. Androgen deprivation therapy suppresses hormone-naïve prostate cancer, but after a certain period of time prostate tumor adapts to survive under low levels of androgens. KLF functions as an AR activator and positively correlates with prostate cancer progression [100]. Liu et al. (2020) established the role of NANOG in prostate cancer stem cell proliferation and its regulation through the SMAD signaling pathway [101]. NANOG silencing decreases phosphorylation events in TGF-β/SMAD signaling components which leads to the inhibition of prostate cancer stem cells proliferation, cell cycle arrest and induction of apoptosis [102]. Overexpression of EZH2 in prostate cancer promotes its progression whereas downregulation of EZH2 inhibits cell proliferation, cell cycle, and invasion in vitro, and tumor reduction in vivo [103]. It is likely that a subset of these molecules together with AQPs might have a better prognostic ability for prostate cancer than a single molecule. Additional studies are required to confirm this hypothesis (Figure 3).

To the best of our knowledge, the prognostic significance of other AQPs including AQP2, AQP4, AQP7, and AQP8 is still enigmatic. Preliminary studies indicate that these AQPs are positively associated with prostate cancer initiation, progression, and recurrence development after therapy. Additional studies are required to establish AQPs as a prognostic biomarker for prostate cancer.

## 6. Conclusions and Future Directions

In conclusion, research over the last decade has shown that AQPs play essential biological roles in cancer, contributing to critical cellular processes such as cell proliferation, migration, and tumor growth. Studies on structural and functional assessment highlight a strong biological relationship between AQPs protein expression, localization, and key biological functions in normal and prostate cancer tissues, where aberrant AQP1, AQP3 and AQP5 expression correlate with tumorigenesis and metastasis. To overcome the prognostic challenges of prostate cancer including stage, grade, and lymph node status additional biomarkers are urgently needed. The research finding from the previous studies indicates having potential that AQPs may serve as prognostic biomarker(s) for prostate cancer. Research investigations found that AQP1 and AQP5 can serve as prognostic biomarker(s) in prostate cancer. Our analysis revealed that AQP1 and AQP3 have prognostic value and may be developed as prognostic biomarkers either alone or together with other identified network proteins. However, additional investigation is still needed. Understanding the unidentified AQP pathophysiology is also essential in order to recognize other AQPs as prognostic biomarkers in prostate cancer.

## Figures and Tables

**Table 1:** Commercially available prognostic biomarkers in prostate cancer.

Category	Commercially Available Tests	Limitations	Ref.
Discriminate aggressive vs. indolent prostate tumors	ConfirmMDx	Additional insights/test/biopsy is needed surrounding high-grade prostate cancer tissue after diagnosis in methylation positive patients.	[10,11]
	Prostate Core Mitomic Test	False negative results in spite high sensitivity, comparable to other biomarker tests.	[12,13]
	Phosphatase and tensin homolog (PTEN) gene	Impact in the detection of prostate cancer due to heterogenic behavior and *PTEN* alterations.	[14,15]
	TMPRSS2-ERG gene fusion	Time-consuming test that requires costly equipment, thus limiting its use in clinical diagnostics.	[16,17]
	ProMark	Biopsy-based test limited to bleeding complication and miss the high-risk areas of prostate tumor.	[18,19]
Improve risk stratification of patients with prostate cancer	Prolaris	Biopsy based test with high probability of missing the high-risk areas of prostate cancer.	[20]
	Oncotype DX	Test not designed to take racial discrimination in account.	[19,21]
	Decipher	Estimate patient risk and influence treatment decisions. It is time taking test with a limitation to require 10 year or more to complete.	[22,23]

**Table 2:** Studies conducted on AQPs in prostate cancer.

AQPs	Cell Lines/Tissue	Methodology	AQPs Expression	Results	Ref.
AQP1	PC-3M	shRNA	Down	Inhibits cell migration	[99]
AQP1	PC-3M	Density-induced pericellular hypoxia and CoCl(2)-induced hypoxia	Up	Hypoxia induces AQP1 mRNA levels via intracellular Ca^2+^, protein kinase C and p38 MAPK signaling pathways.	[66]
AQP3	PC3	Stable knockdown of RalA and overexpression of E-cadherin	Up	AQP3 redistribution inhibits the cell proliferation, enhance cell apoptosis, and suppress motility/invasion.	[83]
	DU-145, PC-3	AQP3-siRNA silencing	Down	Reduces ERK1/2 activation. Inhibited motility/invasion.	[80]
	PC-3, DU145	Cryotherapy of prostate cancer cells, HgCl_2_ as AQP3 inhibitor and AQP3-siRNA silencing	Down	Inhibition of AQP3 increases the sensitivity of prostate cancer cells to cryotherapy.	[60]
	PC-3, DU145, LNCaP, PNT1A Prostate cancer and normal tissue	RT-PCR, IHC	Up	Play a regulatory role in epithelial cell osmolality. Change in the localization of AQP3 in cancer cells as a result of tumorigenesis.	[81]
AQP5	PC-3, LNCaP	AQP5-siRNA	Down	Cell proliferation and migration attenuated.	[94]
	Prostate cancer, prostate epithelium	IHC on a tissue array (*n* = 12,427)	Weak to moderate expression in normal prostate epithelium. Either negative or high expression in prostate cancer	Dichotomous role of AQP5 observed.	[93]
	HPrEC, PC-3, DU145, LNCaP	Immunofluorescence	Up	Differential expression of AQP5 in benign and malignant prostate tissue.	[98]
AQP9	PC-3 Prostate cancer, adjacent tissue	AQP9-siRNA, Western blot, Flow cytometry	Down	Promote apoptosis. Suppressed ERK1/2 phosphorylation, inhibits proliferation, affects the cell motility/invasiveness.	[97]

AQP1, Aquaporin 1; shRNA, Short hairpin RNA; CoCl_2_, Cobalt (II) chloride; MAPK, Mitogen-activated protein kinases; RalA, RAS such as proto-oncogene A; ERK, extracellular signal-regulated kinase; siRNA, Small interfering RNA; HgCl_2_, Mercuric chloride; RT-PCR, Real time polymerase chain reaction; IHC, Immunohistochemistry.

## Data Availability

Not applicable.
